# Methodology for the Review and Update of Nutrient Criteria Underpinning Front-of-Pack Labeling: Application to the Glycemic Index Symbol

**DOI:** 10.3389/fnut.2022.867349

**Published:** 2022-06-09

**Authors:** Carlene Starck, Michelle Blumfield, Kylie Abbott, Tim Cassettari, Jutta Wright, Emily Duve, Alan W. Barclay, Flavia Fayet-Moore

**Affiliations:** ^1^Nutrition Research Australia, Sydney, NSW, Australia; ^2^Riddet Institute, Massey University, Palmerston North, New Zealand; ^3^Accredited Practising Dietitian, Sydney, NSW, Australia

**Keywords:** methodology, front-of-pack labeling, nutrient criteria, Glycemic Index, modeling, nutritional quality

## Abstract

**Background:**

Nutrient criteria underlying front-of-pack food labeling programs can play an important role in improving dietary intakes. Currently, no methodology for the development or update of nutrient criteria has been published, nor the methods used by food regulatory bodies. The scientific publication of methodology outlining the development and update of nutrient criteria underpinning front-of-pack food labeling programs highlighting healthier food choices is needed.

**Objective:**

To develop and provide a globally applicable and transparent methodology for researchers to follow when reviewing existing or developing new nutrient criteria for front-of-pack labeling.

**Methods:**

The Nutrient Criteria Methodology involved five phases: Phase I, the development of guiding principles; Phase II, collection of information for subsequent phases, including a pre-scope of the literature and selection of food composition database(s) for modeling; Phase III, literature review of all possible nutrients relevant to the nutrient criteria; Phase IV, database modeling to set quantitative limits for each selected nutrient; Phase V, assessment of the criteria against an established nutritional quality assessment tool. As an example, the methodology was applied to the update of the GI Symbol Product Eligibility and Nutrient Criteria (PENC).

**Results:**

A comprehensive and replicable methodology, based on best practice protocols and ensuring both scientific credibility and practicality of use by industry, was developed. Application of the five phases of the methodology to the GI Symbol PENC highlighted the ability of the methodology to uncover nutritional measures currently missing in many nutrient criteria for front-of-pack food labeling programs and other national food labeling systems, such as glycemic load and the unsaturated to saturated fat ratio. Foods achieving the PENC had a higher Health Star Rating than foods not achieving the PENC.

**Conclusion:**

Our Nutrient Criteria Methodology can be applied to the development and update of global nutrient criteria underpinning front-of-pack food labeling programs. Further research into the implementation of additional nutritional measures found to be important for human health is recommended, with the goal of the prevention of diet-related disease.

## Introduction

Nutrient criteria can play an important role in improving population health by classifying or ranking foods according to their nutritional composition (1). As an underlying factor in the development of front-of-pack labeling (FoPL) systems, such as Australia's Health Star Rating (HSR) (2), the Glycemic Index (GI) Symbol (3), and the Guiding Stars intervention in the US (4), all of which aim to highlight healthier food products within a food category for all population groups, nutrient criteria assist consumers in making healthier food choices (1, 3, 5), and are considered a valuable tool in the prevention of diet-related chronic disease (6). However, no methodology for the development of new nutrient criteria by food regulatory bodies has been published, and the detailed methods used by these bodies are rarely disclosed. As this can result in inconsistencies across the nutrient criteria used to support consumers in making healthier choices, the scientific publication of methodology outlining the development of nutrient criteria by regulatory bodies is needed.

As our understanding of food, nutrients and health continues to evolve, the regular review and update of nutrient criteria is important to ensure the ongoing improvement of population's diet and health. While a formal review of Australia's HSR System was recommended after 5 years of implementation to determine its performance and options for enhancement (7), there is still no method detailing the update of existing nutrient criteria readily available. Nutrition science and the food supply are both constantly evolving, and the provision of a methodology for the update of nutrient criteria that is both scientifically credible and aligns with the needs of the food industry and population health, is required.

Worldwide, inconsistency surrounds the nutritional components that underpin nutrient criteria and how these are regulated within food labeling systems, potentially creating confusion for consumers and increasing product costs if re-labeling is required, especially when products are imported into other nations with different food regulatory systems (3). This inconsistency is amplified when one considers the wide spectrum of methods used to make nutrition, health, and related claims; in addition to FoPL systems, these methods include nutrition content claims (presence or absence of a nutrient), health claims (a food or property of the food has a health effect), and endorsements (nutrient content or health claims made with the permission of an endorsing body) (3). FoPL systems have different nutritional measures that are designed to rate the overall nutritional profile (i.e., ‘healthiness') of packaged foods (8), and perform differently in the context of increasing the nutritional quality of food choices (8, 9). Country specific FoPL regulations have resulted in foods manufactured in some countries being required to meet a strict set of nutrient criteria to make a nutrition, health or related claim, while in other countries, nutrient criteria for the same claims are lacking (3). For example, while the strict nutrient criteria underpinning the GI Symbol ensures that healthier low GI food products are highlighted in Australia and New Zealand, the regulatory approach to GI claims on food labels is inconsistent globally, creating confusion around the selection of healthier choices, particularly in those with or at risk of diabetes (3). Furthermore, some FoPL systems developed by the private sector have been suggested to be used for marketing purposes and potentially mislead consumers (8). To address these differences, reduce confusion and ensure access to healthy food products, the methodology applied to the development and update of nutrient criteria should be both transparent and standardized.

Here, we propose a Nutrient Criteria Methodology to provide a transparent process for researchers to follow when developing or reviewing existing new nutrient criteria. The aim of this study was to develop a Nutrient Criteria Methodology that could be applied globally. The first part of this paper describes the methodology that was developed. Next, we present the results for an application of the methodology using the revision of the GI Symbol Product Eligibility and Nutrient Criteria (PENC). Finally, potential enhancements to the methodology that may advance its use and further considerations for practical application are discussed.

## Methods

**Figure 1:**
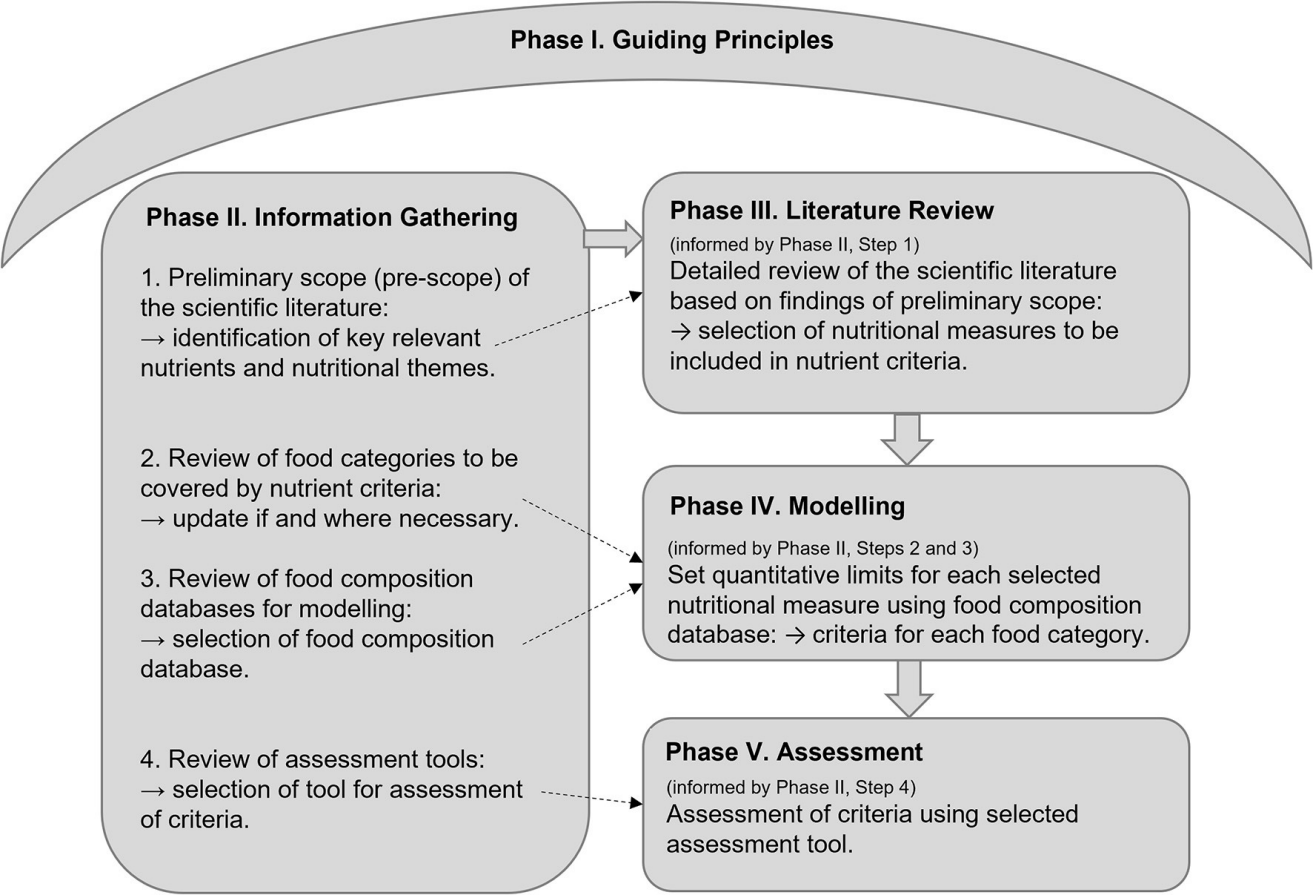
Summary of the nutrient criteria methodology: The five Phases. Phase I, the development of guiding principles to underpin decision-making in each subsequent phase. Phase II, information is collected to inform subsequent phases by conducting: (1) Pre-scope of the scientific literature to inform Phase III; (2) Update of food categories covered by the nutrient criteria to inform Phase IV (if applicable); (3) Review of food composition databases to inform Phase IV; and (4) Review of nutritional quality assessment tools for the updated criteria to inform Phase V. Phase III, literature review of all possible nutrients relevant to the nutrient criteria to inform nutrient selection in the criteria. Phase IV, food modeling to set quantitative limits for each selected nutrient, by food category. Phase V, assessment of the criteria against an established nutritional quality assessment tool.

The Nutrient Criteria Methodology includes five phases and is summarized in Figure 1. Each of the five phases will require some modification, depending on the specific characteristics of the nutrient criteria to be developed or updated, such as the objectives of the front-of-pack symbol associated with the nutrient criteria, the nutritional components included, and the nutritional information available for modeling. While it is therefore not feasible to produce time estimates for each phase, the detail provided is intended to guide timing estimations. In line with the protocol applied to the HSR, it is recommended that nutrient criteria should be reviewed and updated every five years (7).

### Phase I. Guiding Principles

To ensure that the Nutrient Criteria Methodology are evidence-based, relevant, and transparent, a set of guiding principles, specific to the objectives of the nutrient criteria development, need to be established. These guiding principles are designed to inform decision-making in each subsequent phase of the process to align the nutrient criteria with its overall objectives. Guiding principles can be developed based on consultation with academia, industry, and any other relevant stakeholders, by asking the following questions:

- What are the key objectives of the nutrient criteria in general?
- What are the key objectives of the nutrient criteria in question?
- How can we ensure that the nutrient criteria are scientifically credible?
- What needs to be considered from a food industry perspective, to ensure the criteria have practical relevance?

### Phase II. Information Gathering

In Phase II, information is collected to inform all subsequent phases, according to four steps:

#### Step 1. Perform a Preliminary Literature Scope (Pre-scope)

The purpose of the pre-scope is to inform the direction of the literature review (Phase III) that will determine the nutritional measures for inclusion in the nutrient criteria. While a multitude of different nutritional measures might be possible, not all will be relevant to the nutrient criteria in question, the population group targeted by the nutrient criteria, or backed by robust scientific evidence. The pre-scope aims to uncover those nutritional measures that are most relevant to the nutrient criteria in question and pertinent to human health.

The following steps can be taken, and are based on a targeted simplification of the Cochrane rapid review methodology to reduce time and uncover high level information (10):

1. Determine the search strategy.
  - Develop research questions to identify relevant nutritional themes and the health/dietary indicators that will uncover the level of significance of each nutritional theme to be measured.
  - Develop search terms based on keywords within each of the research questions and the overall objective(s) of the nutrient criteria in question (as identified in Phase I).
  - Select at least one relevant electronic scientific database (e.g., Pubmed, Embase, Scopus) to identify key scientific literature, and 1 secondary database (e.g., Google Scholar) to identify relevant gray literature (e.g., information from regulatory bodies).
2. Execute the search strategy in the selected electronic databases.
3. When screening studies, prioritize the most recent review articles (particularly systematic literature reviews (SLRs) if available) to obtain the most up to date scientific understanding on the topic.
4. Extract summary-level data from selected key papers and organize information by nutritional theme and health/dietary indicators.
5. Prioritize the nutritional themes and health/dietary indicators identified as having the greatest impact on the nutrient criteria and on human health.

#### Step 2. Determine the Full Range of Food Products and Food Categories

Due to continuous advancements in the food industry and consumer demand, a complete list of food categories is required to reflect all current and emerging food categories applicable to the specific nutrient criteria. As food categories may differ significantly in nutritional characteristics (e.g., breads/cereals vs. milk/dairy products), specific nutrient criteria will be required for each nutritionally distinct food category. Determination of the full range of food products and food categories may be carried out in three steps:

Comparison of the food categories covered by the current nutrient criteria (if applicable) with present-day food products, followed by the removal or addition of any food products/categories that are no longer relevant or missing, respectively.

Consultation with industry stakeholders to ensure all existing and emerging food products related to the nutrient criteria in question are captured.

Organization of all relevant food products with similar nutrient profiles into food categories specific to the nutrient criteria being developed.

#### Step 3. Select a Food Composition Database for Statistical Modeling (in Phase IV)

The purpose of statistical modeling is to develop quantitative limits or thresholds for each nutritional measure within each food category, resulting in a set of nutrient criteria for each food category. To facilitate this process, a database containing a comprehensive list of food products within each relevant food category and the nutritional composition of each food product is required; this is a food composition database. Selection of the food composition database to be utilized in the nutrient criteria development by asking the following questions:

- Is the nutrient information for the majority of foods used by the target population readily available?
- Does the database contain up-to-date information accurately reflecting the current food supply (i.e., recently developed or updated)?
- Is it representative of the full scope of food products within each food category (i.e., comprehensive)?
- Does it provide a wide range of nutritional information?
- Does it enable easy calculation and extraction of the data? (i.e., file format)
- Has it been successfully used in previous modeling projects or published research?

#### Step 4. Review and Select a Nutritional Quality Assessment Tool

Quantitative assessment is required to confirm the validity and functionality of the nutrient criteria (e.g., ability to select the healthiest foods within each food category). All potential assessment tools measuring nutritional quality should be considered. The selection of an assessment tool may be based on the:

- Correlation of food categories covered by the assessment tool with those in the nutrient criteria;
- Correlation of nutrients and other food-based nutritional measures (e.g., fruit and vegetable component) in the assessment tool and nutrient criteria;
- Availability of nutritional information required by the assessment tool in the food composition database selected for modeling.

### Phase III. Literature Review

In Phase III, a literature review is performed to obtain comprehensive evidence for the health and diet quality indicators related to each nutritional measure, as identified in Phase II, Step 1. While the pre-scope (Phase II, Step 1) provides a general understanding of the relevant scientific evidence, a detailed literature review is required to obtain a level of data sufficient to make robust decisions on which nutritional measures should be included within the nutrient criteria, including quantitative data that can be applied to the modeling, if applicable.

To ensure the literature review is conducted with the most efficient use of resources, a protocol based on a modification of the Cochrane rapid review methodology can be applied (10) and involves the following 10 steps:

1. Refine the research questions and search terms based on the nutritional themes and health/dietary indicators identified in the pre-scope (Phase II, Step 1).
2. Develop the search strategy, and select at least two electronic scientific databases and one secondary database to identify relevant gray literature.
3. Use the PICOS eligibility criteria to determine study inclusion, prioritizing SLRs and RCTs if available (11).
4. Execute the search strategy in the selected electronic databases.
5. Screen studies for eligibility by title and abstract. Retrieve full texts for all studies that appear to meet the eligibility criteria, then assess full-texts for relevance.
6. Extract data from included studies into a simplified data extraction table or database spreadsheet (example template in Supplementary Table S1).
7. Summarize the evidence for each nutritional measure and health/dietary indicator.
8. Critically appraise the quality, strength, and consistency of the evidence for each nutritional measure per each health/dietary indicator using a quality appraisal tool.
9. Consider the feasibility of each nutritional measure for use by the food industry via consultation with industry stakeholders.
10. Integrate findings from steps 8 and 9 to determine the nutritional measures with the highest scientific credibility, and feasibility for use by industry, and thus suitable for inclusion in the nutrient criteria.

### Phase IV. Modeling

In Phase IV, statistical modeling using the food composition database (Phase II, Step 3) is conducted. The modeling aims to determine the quantitative thresholds for each nutritional measure in the criteria, for each relevant food category, and is guided by results from the literature review and the purpose of the nutrient criteria.

Prior to the modeling, the selected food composition database should be checked and edited, if necessary, to remove all duplicates, foods missing relevant nutritional information, and food categories not covered by the nutrient criteria.

Modeling involves three steps:

#### Step 1. Combine the Food-Based Recommendations for Each Relevant Nutritional Measure

These include country-specific regulations for nutrition, health or related claims and food-level recommendations from dietary guidelines or global regulatory bodies. For example, if the nutritional measure is protein, and the criteria is being developed in Australia or New Zealand, then the Food Standards Australia New Zealand (FSANZ), then the nutrient content claim(s) criteria for food to contain or be a good source of protein would be included.

#### Step 2. Select the Most Relevant Nutritional Measures for Each Food Category

In the food composition database, nutritional measures can be selected for inclusion in the criteria by assessing the distribution (e.g., mean, median, minimum, maximum, etc.…) of each nutritional measure compared to the:

Aggregated food-based recommendations for that nutritional measure. For example, if the nutritional measure is protein and the food category is ready-to-eat meals, then the distribution of protein levels in all food products included in the ready-to-eat meals food category would be assessed against the levels identified in Phase IV, Step 1. It would include the levels necessary to make a nutrient content claim for protein.

- Presence of outliers, defined as values that were abnormally high for nutrients considered to have a negative effect on health (e.g., sodium, saturated fat).
- Findings from the pre-scope of the literature carried out in Phase II.
- A nutritional measure can be confirmed for a food category when there are:
- Food products containing higher or lower than the recommended limits, depending on whether the nutritional measure is considered detrimental or beneficial for health, respectively; or,
- The presence of outliers (as defined above) for that nutritional measure; or,
- Evidence that a nutritional measure should be included within a specific food category.

#### Step 3. Quantify the Limits for Each Nutritional Measure Within Each Food Category

Limits are set based on the current nutrient criteria limits or identified food-based recommendations, as identified in Phase IV, Step 1 and 2. If no pre-existing target or recommendation exists, then the limit may be set to remove outliers. If no outliers are present, then the limit can be based on the mean or median level of that nutrient, within each food category. Each limit imposed should be tested for stringency and appropriateness by assessing the nutritional composition and ingredients list of foods that ‘achieved' vs. ‘did not achieve' the criteria for healthiness (i.e., containing a high level of nutrients and ingredients recommended to include in the diet, such as dietary fiber, and a low level of nutrients and ingredients recommended to limit, such as sodium and saturated fat). A quantitative limit may be selected when foods that ‘achieve' the criteria have a healthier nutritional composition compared to those that ‘do not achieve' the criteria.

### Phase V. Quantitative Assessment of the Nutrient Criteria

The purpose of the quantitative assessment is to quantitatively determine the ability of the front-of-pack nutrient criteria to perform the intended task, as defined within the guiding principles (e.g., the ability to select healthier foods within each food category). Assessment can be carried out over three steps:

#### Step 1. Assessment of the Criteria Using the Selected Nutritional Quality Assessment Tool

Depending on the resources available, two approaches have been developed to assess the validity of the nutrient criteria:

Comprehensive assessment (optimal approach): A random selection of foods that ‘achieve' and ‘do not achieve' the nutrient criteria from every food category applicable to the nutrient criteria.

Restricted assessment: Selection of a smaller number of food categories, representative of the core food groups (e.g., a food category such as breakfast cereals to represent grains and cereals; a food category such as dairy milk to represent dairy).

For each selected food, the assessment tool should be applied and the nutritional quality score calculated. The mean score (± SD) for foods that ‘achieve' and ‘do not achieve' the nutrient criteria are determined and compared using the student's *t*-test for statistical significance *(p* < 0.05). Statistical analyses can be performed in a range of statistical analysis software (e.g., SPSS, MS Excel, etc).

#### Step 2. Secondary Assessment of the Nutrient Criteria Against Foods Currently Meeting the Nutrient Criteria (if Applicable)

For many existing nutrient criteria, a portfolio of foods currently covered by the criteria are available. If the data are available, a secondary assessment can be performed. All foods that are currently certified as meeting the nutrient criteria can be assessed against the updated criteria and their scores calculated. The mean assessment score (± SD) for food products that ‘achieve' vs. ‘did not achieve' the updated nutrient criteria can then be determined and compared using the student's *t*-test for statistical significance (*p* < 0.05). Statistical analyses are then performed in statistical analysis software.

#### Step 3. Stakeholder Review of Updated Nutrient Criteria

To ensure both scientific credibility and alignment with the constraints of the food industry and consumer demand, the updated nutrient criteria may be reviewed by independent academic and industry stakeholders. Findings from Phase V are then integrated into the criteria where necessary to improve performance.

### Application of the Nutrient Criteria Methodology: Example Using the GI Symbol Product Eligibility and Nutrient Criteria (PENC)

The GI Symbol is a Certification Trademark established by the GI Foundation of Australia that can be applied to carbohydrate-containing foods that have both a GI ≤ 55 and meet the PENC (3). The GI Symbol was established in 2002 to address confusion around the selection of healthier carbohydrate choices, including for people with diabetes, by flagging healthier carbohydrate foods and food products. The PENC was last updated in 2015 and in 2021, the Nutrient Criteria Methodology was applied to update the GI Symbol PENC.

## Results

The update of the GI Symbol PENC using the Nutrient Criteria Methodology is described below as an example of its application.

### Phase I. Guiding Principles

The guiding principles developed for the GI Symbol PENC update were:

- Based on current, authoritative, and robust peer reviewed evidence.
- Developed with consideration to the specific requirements and/or constraints of its users.
- Easy for users to understand and implement.
- PProvision of thought leadership.
- Application only to foods that provide a source of available carbohydrate in the diet, as defined by the International Standard for determination of the glycemic index, ISO 26642:2010 (12).
- Designed to provide guidance on choosing healthier food choices within a food group or category, rather than being applicable to only core or unprocessed foods.

### Phase II

#### Step 1. Perform a Preliminary Literature Scope (Pre-scope)

To review the PENC, the pre-scope aimed to answer the following questions:

- What is the most recent scientific understanding surrounding carbohydrate quality?
- What related nutritional measures currently exist and what is the recent scientific understanding on these?
- What (a) health and (b) dietary outcomes are most relevant to the nutrient criteria?

A targeted literature scope was conducted on the 21 May 2021, via the Pubmed and Google Scholar databases, and the Google search platform. The most recent review articles for each nutritional theme (including SLRs) were prioritized to obtain the most up to date scientific understanding.

The focus areas identified by the pre-scope, to be further investigated in the subsequent literature review (Phase III), are presented in Table 1. Two areas were excluded from further review; and these were GI (since a GI ≤55 is an inherent requirement of all food products assessed by the GI Symbol PENC) and resistant starch, since this would be included as part of the evidence obtained for dietary fiber.

**Table 1:** Summary of focus areas identified by the pre-scope.

**Research question**	**Focus areas**
1. Carbohydrate quality	Glycemic load, Fiber, Whole grains, Starch
2. Related nutritional measures	Sugars (total, added, and free), Sodium, Fatty acid profile, Protein, Energy, Potassium, Processing level
3. (a) Health outcomes	Cardiovascular disease, Type 2 diabetes mellitus, Metabolic syndrome, Cancer, Risk factors for each health outcome
(b) Dietary outcomes	Diet quality, Nutrient intakes.

#### Step 2. Determine the Full Range of Food Products and Food Categories

The original GI Symbol PENC included 39 food categories. The update of these food categories resulted in 17 new food categories being created, primarily from additional carbohydrate-containing food products entering the market, including toddler foods and frozen potato products. A list of the final 56 food categories selected for the PENC update is provided in Supplementary Table S2.

#### Step 3. Select the Food Composition Database for Statistical Modeling (in Phase IV)

The assessment of food composition databases for the PENC update is summarized in Supplementary Table S3. Potential food composition databases were reviewed via a search of the scientific literature (via PubMed) and the Google search engine on the 24^th^ May 2021, as well as in consultation with stakeholders. The food composition database selected was the FoodSwitch database, managed by the George Institute for Global Health (13). The FoodSwitch database is comprehensive, relevant to the current food industry, and has been used extensively for modeling in previous studies. However, it is available at a cost and the version accessed did not contain data for added sugars and trans fats. The AUSNUT database (14), which is freely available for download, was selected for the modeling of added sugars and trans fats. This was not selected for the full modeling due to the last update being a decade old (2011–2012). The use of both databases provided the ability to assess all potential nutritional measures for all food categories, maintaining the use of the most up-to-date food product data for the majority of nutritional measures used in the nutrient criteria update.

#### Step 4. Review and Selection of Nutritional Quality Assessment Tools

The nutritional quality assessment tool selected for use was the Health Star Rating (HSR) system (2). The HSR is widely used in both Australia and New Zealand, correlated well with the nutrients included in the updated PENC, and has been routinely used in conjunction with the FoodSwitch database (7). A summary of the nutritional quality assessment tools considered for the update of the GI Symbol PENC is provided in Supplementary Table S4.

### Phase III. Literature Review

Based on the findings of the pre-scope (Phase II, Step 1), the research questions underpinning the literature review were:

1. What measures of carbohydrate quality should be included in the update of the GI Symbol PENC?
2. What related nutritional measures, outside of carbohydrate quality, should be included in the update of the GI Symbol PENC?

Literature searches were conducted on the 15 and 16th June, 2021, using the Medline, PubMed, Cochrane Library, and Google Scholar databases. A secondary targeted search in Google Scholar was also conducted to identify the most recent key position papers on each nutrient or nutritional measure from reputable organizations (e.g., World Health Organization (WHO), Food Standards Australia New Zealand (FSANZ), and the Heart Foundation of Australia). Inclusion and exclusion criteria were defined by the PICOS framework (11). All populations were considered, with no restrictions on age, sex, or ethnicity. Nutritional interventions and health and dietary outcomes were limited to those identified in the pre-scope (Table 1). There were no exclusions on comparators. SLRs and SLRs with meta-analyses (MAs) of randomized controlled trials (RCTs) and cohort studies were prioritized, with individual RCTs and cohort studies only considered when SLRs and MAs were not available. Cross-sectional studies were only sourced if no RCTs or cohort studies were available. Literature published from 2015 onward was selected based on the previous criteria update. The evidence selected for each nutritional measure was appraised for quality according to the National Health and Medical Research Council hierarchy of evidence framework (15). Consultation with industry stakeholders was carried out to determine feasibility of each nutritional measure for use by industry.

Results from the literature review identified two measures of carbohydrate quality and ten related nutritional measures that should be applied to the update of the GI Symbol PENC (Table 2). A summary of the literature review findings is presented in Supplementary Methods S1, Part A.

**Table 2:** Summary of nutritional measures selected for inclusion in the updated PENC.

**Nutritional measures**	**To be included in update of GI symbol PENC**
Carbohydrate quality	Glycemic load, Fiber, Carbohydrate to fiber ratio
Related nutritional measures	Total sugars, Added sugars, Sodium, Saturated fat, Unsaturated fat to saturated fat ratio, Trans fat, Protein

*PENC, Product Eligibility and Nutrient Criteria; GI, Glycemic Index*.

### Phase IV. Modeling

The FoodSwitch database was updated to remove all duplicates, foods missing relevant nutritional information, and foods not covered by the nutrient criteria.

#### Step 1. Combine the Food-Based Recommendations for Each Relevant Nutritional Measure

Food-based recommendations relevant to the PENC included the Healthy Food Partnership (HFP) reformulation targets (16) and the FSANZ nutrient content claims (17); plus additional food-level recommendations from health authorities were included where identified. A summary of the aggregated food-based recommendations from HFP reformulation targets, the FSANZ nutrient content claims, and additional guidelines identified in the scientific and gray literature are detailed in Supplementary Table S5.

#### Step 2. Select the Most Relevant Nutritional Measures for Each Food Category

**Figure 2:**
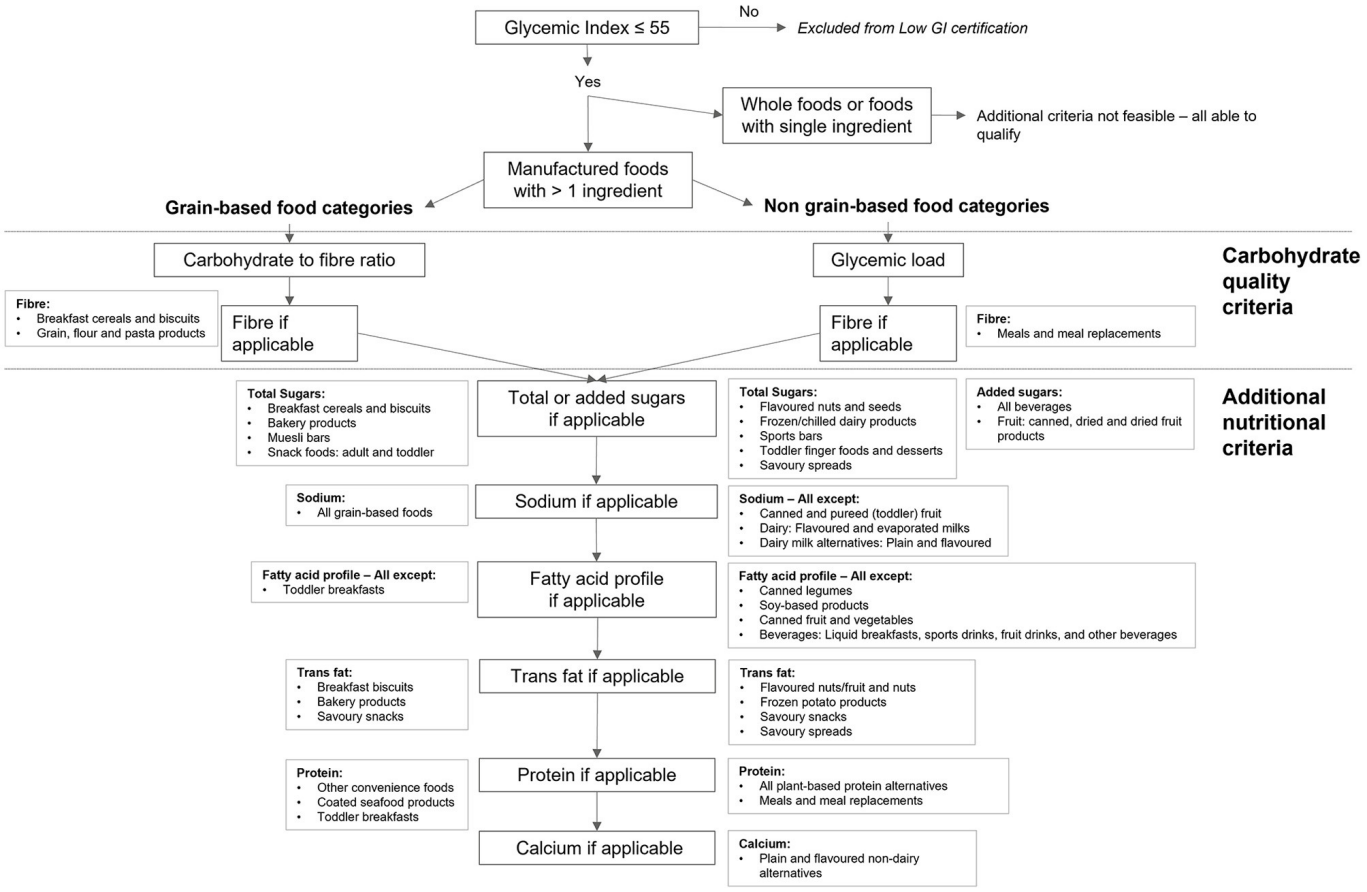
Overview of the nutritional measures determined for each food category covered by the GI Symbol PENC. All foods must have GI ≤ 55 to be considered for the GI Symbol. Reformulation was not considered feasible for whole foods of foods with a single ingredient; these foods were not subject to additional criteria. Manufactured foods with more than one ingredient were separated into grain-based and non-grain-based food categories. The criteria for grain-based food categories included the carbohydrate to fiber ratio and absolute fiber if necessary; the criteria for non-grain-based food categories used glycemic load in place of the carbohydrate to fiber ratio. All food categories were subject to criteria for total or added sugars, sodium, fatty acid profile including trans fats, protein, and calcium, as determined necessary by the modeling. The same criteria apply to food categories meeting lower GI criteria (GI 25% lower than that of a reference food).

A flow-chart depicting the nutritional measures determined for each food category is shown in Figure 2. Each food category was first classified according to the degree to which reformulation was feasible. Reformulation was considered feasible for manufactured foods with more than one ingredient. Products for which reformulation was not feasible were not subject to additional criteria; these included whole foods (e.g., fresh fruit) or single ingredient foods (e.g., sugars). Of the 56 food categories, seven were deemed to be not feasible for reformulation, resulting in the modeling of 49 individual food categories. Carbohydrate quality measures were applied based on whether a food category was grain based (carbohydrate to fiber ratio) or not (glycemic load). Following the application of carbohydrate quality measures, additional nutritional measures were applied to both grain-based and non-grain-based food categories, on a food category specific basis. Based on stakeholder recommendation, a minimum calcium limit was also applied to food categories intended to serve as dairy milk analogs.

#### Step 3. Quantify the Limits for Each Nutritional Measure Within Each Food Category

**Figure 3:**
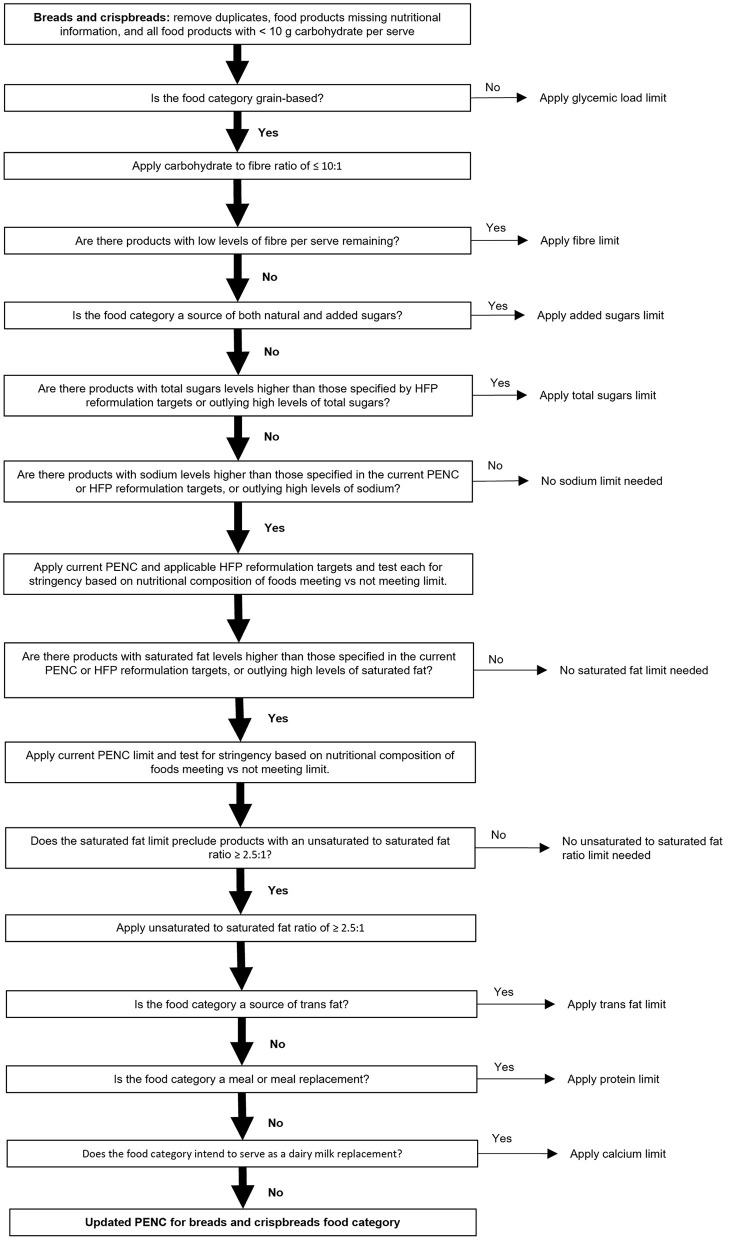
Overview of the specific step-wise modeling strategy for the ‘breads and crispbreads' food category. Where a yes/no option existed, only the selection (yes/no; vertical path and shown by a solid black arrow) followed for breads and crispbreads has been described in detail.

The full GI Symbol PENC containing limits for each nutritional measure within each food category is freely available via the ACCC and GI Foundation websites. An example of the step-by-step modeling process specific to the ‘breads and crispbreads' food category is provided in Figure 3. Additional detail regarding the nutritional measures and limits selected per each food category is provided in Supplementary Methods S1, Part B.

### Phase V. Quantitative Assessment of the Nutrient Criteria

#### Step 1. Assessment of the Criteria Using the Selected Nutritional Quality Assessment Tool

Two food categories were selected to assess the update of the GI Symbol PENC against the HSR. Selection was based on each food category containing the largest number of representative products within their core food group but having distinct nutritional characteristics; these were ‘breads and crispbreads' and ‘yogurts - sweetened'. While the optimal approach would include HSR assessment of all 56 food categories, a reduced approach was used to reduce time and cost. The selected food categories were deemed to be predominant in the diet and found to be representative of the majority of other food categories in their core food group. For each of the selected food categories, twenty products from within the FoodSwitch database were randomly selected; ten meeting the PENC and ten not meeting the PENC.

Findings for the HSR comparison of products that ‘achieved' vs. ‘did not achieve' the PENC are summarized in Table 3. For the ‘breads and crispbreads' food category, products achieving the updated GI Symbol PENC had a significantly higher HSR than products not achieving the updated PENC (3.9 ±0.49 vs. 2.1 ±0.62, p<0.001). For the ‘yogurts – sweetened' food category, there was a trend for products achieving the updated GI Symbol PENC to have a higher HSR than products not achieving the updated GI Symbol PENC (3.6 ±1.04 vs. 2.5 ±1.31; *p* = 0.054). Within ‘yogurts – sweetened', some products meeting the updated PENC had a low HSR, while other products not meeting the updated PENC had a high HSR (data not shown).

**Table 3:** HSR assessment of updated GI Symbol PENC for selected FoodSwitch products within the ‘breads and crispbreads' and ‘yogurt – sweetened' food categories, and the current GI Symbol portfolio of foods.

**Food grouping**	**HSR, mean (SD)**		
	**Achieve PENC**	**Do not achieve PENC**	***P*-value^a^**
Breads and crispbreads	3.9 (0.49)	2.1 (0.62)	<0.001
Yogurts - sweetened	3.6 (1.04)	2.5 (1.31)	0.054
Current GI Symbol portfolio	3.1 (1.67)	1.5 (1.52)	0.004

a *For statistical significance, p < 0.05*.

*HSR, Health Star Rating; PENC, GI Symbol Product Eligibility and Nutrient Criteria*.

#### Step 2. Secondary Assessment of the Nutrient Criteria Against Foods Currently Achieving the Nutrient Criteria (if Applicable)

Of the 163 food products currently carrying the GI Symbol, 151 (93%) achieve the update of the GI Symbol PENC (data not shown). The HSR of products carrying the GI Symbol and that satisfied the updated PENC were higher compared to those that did not satisfy the updated GI Symbol PENC (HSR 3.1 vs. 1.5; *p* = 0.004) (Table 3).

#### Step 3. Stakeholder Review of Updated Nutrient Criteria

Key academic and industry stakeholders were sent the update of the GI Symbol PENC for review and feedback. Stakeholder feedback was independently assessed against the guiding principles and integrated into the updated criteria where appropriate to enhance the performance of the nutrient criteria. For example, it was suggested that calcium limits were applied to the dairy milk alternatives (summary of feedback provided in Supplementary Results S1).

## Discussion

A comprehensive methodology for the review of existing or development of new nutrient criteria for FoPL systems was developed, with the GI Symbol PENC update utilized as an example of its application. The methodology was established based on best practice protocols and aimed to ensure both scientific credibility and practicality of use by industry. Application to the GI Symbol PENC highlighted the ability of the methodology to uncover nutritional measures currently missing in many nutrient criteria and other national food labeling systems, such as glycemic load and the ratio of unsaturated to saturated fat. These nutritional measures warrant consideration for incorporation into national nutrient criteria and emphasize the need for the ongoing update of nutrient criteria used to guide population health as nutrition science continues to evolve.

Despite the many nutrient criteria that exist and the fact that many have been subject to periodic updates, no transparent, standardized methodology for the development nor update of nutrient criteria was identified. The reason for this is not clear. While the 5 year review of the HSR System, published in 2019 (7), provided detailed results as well as an overview of the review process, it did not include a detailed description of the methodology used. In comparison, the methodology used for the development of HFP reformulation targets was described in detail, particularly for the selection of food categories requiring reformulation, and there was a short description of the modeling methodology used to set reformulation targets (18). However, there was no step-wise description of the methodology that would allow future researchers to reproduce the methods used. Similarly, while the OfCom nutrient profiling model underpinning the Nutri-Score food labeling system has been explained and assessed over a number of different publications (19–21), no step-wise methodology was identified either. Some nutrient criteria developed by the food industry have been criticized as being targeted toward marketing purposes, with the potential to be misleading if applied to products with poor nutritional profiles (8). To unify efforts in improving population health in a credible manner, our goal was to provide a clear and rigorous pathway, based on established methods, that all researchers looking to develop, or update, any nutrient criteria could follow.

As our understanding of nutrition science and the food supply are constantly evolving, the alignment of nutrient criteria with both science and industry will ultimately support the selection of healthy foods by the public. While the most recent nutrition science may highlight the most optimal targets for health, food product development is also bound by what is feasible within the constraints of food manufacturing, and consumer acceptance of food products. If a nutrient criterion is so stringent that targets cannot be met by the food industry, certification will not be enabled for any food products within a food category, and there will be no guidance for consumers. Similarly, if a set of nutrient criteria are too relaxed, too many products will be able to meet the criteria, undermining the overall purpose of the criteria. A balance between nutrition science and food manufacturing can be achieved using the methods described here, notably through: review of the most recent and relevant scientific literature, based on an established protocol (10); modeling of the predominant nutritional measures identified against a comprehensive database of commonly consumed foods; and, consultation with key academic and industry stakeholders throughout the entire process.

The application of the proposed methodology to the GI Symbol PENC uncovered several nutritional measures that show importance for population health but are lacking in many nutrient criteria, as well as other national food labeling systems. For example, while there was correlation between foods meeting the PENC and higher scores according to the HSR for the majority of food products, this correlation was not apparent on assessment of the ‘yogurts – sweetened' food category. A predominant reason underpinning this result was the difference in the nutritional measures included in the PENC for dairy products compared to the HSR, particularly glycemic load (which excluded some high HSR food products from meeting the updated PENC) and the unsaturated to saturated fat ratio (which permitted some low HSR food products to meet the updated PENC). As both glycemic load and the unsaturated to saturated fat ratio were identified as key measures for health and diet quality, consideration for their inclusion in national food labeling systems is suggested. Another predominant finding of the literature review was the importance of carbohydrate quality for health, for which multiple markers were identified, including glycemic load and the carbohydrate to fiber ratio. Although a number of carbohydrate quality indices, combining multiple carbohydrate quality markers, have been published, no consensus was identified; highlighting the need for development of an easy-to-use carbohydrate quality index that can be incorporated into nutrient criteria. Some nutritional measures showing a significant link to health were not able to be included in the PENC due to methodological or industry constraints. For example, whole grain intake was found to correlate inversely with type 2 diabetes (22, 23) but was excluded from the PENC due to a lack of information about wholegrain content in existing food composition databases, and hence, the inability to be applied to the modeling. While an increased sodium to potassium ratio was identified as a key risk for hypertension and obesity (24, 25), potassium was also excluded because it is not currently mandated on nutrition information panels in Australia and New Zealand. Further investigation into methods that would enable nutritional measures such as these to be included in nutrient criteria may play an important role in the prevention of diet-related disease. A limitation of the Nutrient Criteria Methodology presented is its application to only one nutrient criteria at time of publication; the GI Foundation's PENC as presented here as an example of its application. While each step of the methodology was developed based on established best practice protocols to increase credibility and confidence in performance, application to an increased number of nutrient criteria will be necessary to ensure ongoing refinement and uncover any specific considerations for different groups of nutrient criteria. The protocol used for assessment of the PENC (Phase V) was a shortened protocol that did not cover all food categories addressed by the PENC due to resource constraints. While the selection of the assessed food categories was carried out to optimize both sensitivity (food categories that were highly populated) and reduce bias toward specific nutritional measures (food categories differing in nutritional profile), the gold standard approach would be to assess and compare all foods meeting and not meeting the criteria in all food categories. Depending on the nutrient criteria in question, this approach would likely require significant resources including time and monetary investment.

The Nutrient Criteria Methodology presented has a number of strengths. The methodology is holistic in nature, integrating multiple systems and processes that build on both scientific and industry expertise. Each phase of the protocol has been described in a step-wise manner and applied to a well-known FoP food labeling system to ensure familiarity and ease of reproducibility. Each phase was developed using established best practice methods, increasing potential for application to a wide range of different nutrient criteria.

## Conclusion

This study describes a Nutrient Criteria Methodology, based on best practice protocols, that can be applied to the development and revision of global nutrient criteria, in particular from FoPL systems. This step-by-step framework has been applied to the GI Symbol PENC as an example, to increase the performance, credibility, consistency, and transparency of the GI Symbol and to ensure its reproducibility. The methodology provides a clear pathway for researchers looking to develop or update, any nutrient criteria, and will assist in aligning nutrient criteria and food labeling regulations for the practical promotion of global human health in an economical, straightforward, and standardized manner. Further research into the implementation of additional nutritional measures found to be important for human health but not applicable to nutrient criteria at the current time is recommended and may play an important role in the prevention of diet-related disease. Keywords: methodology, Front-of-pack labeling, nutrient criteria, Glycemic Index, modeling, nutritional quality.

## Data Availability Statement

The data analyzed in this study is subject to the following licenses/restrictions: the FoodSwitch database used for modeling is available on application to the George Institute for Global Health.

## Author Contributions

CS organized the modeling database, performed the data and statistical analysis, and wrote the first draft of the manuscript. All authors contributed to the study concept and design, and manuscript revision. All authors contributed to the article and approved the submitted version.

## Funding

This project has been funded by a research grant from Glycemic Index Foundation, Australia.

## Conflict of Interest

The authors declare that the research was conducted in the absence of any commercial or financial relationships that could be construed as a potential conflict of interest.

## Publisher's Note

All claims expressed in this article are solely those of the authors and do not necessarily represent those of their affiliated organizations, or those of the publisher, the editors and the reviewers. Any product that may be evaluated in this article, or claim that may be made by its manufacturer, is not guaranteed or endorsed by the publisher.

## References

[1] Dunford EK Huang L Peters SAE Crino M Neal BC Ni Mhurchu C. Evaluation of alignment between the health claims Nutrient Profiling Scoring Criterion (NPSC) and the Health Star Rating (HSR) nutrient profiling models. Nutrients. (2018) 10:1065. 10.3390/nu10081065 30103402 PMC6115993

[2] Health Star Rating System: Australian Government. Available online at: http://www.healthstarrating.gov.au/internet/healthstarrating/publishing.nsf/Content/home (accessed May 14, 2021).

[3] Barclay AW Augustin LSA Brighenti F Delport E Henry CJ Sievenpiper JL. Dietary glycaemic index labelling: a global perspective. Nutrients. (2021) 13:3244. 10.3390/nu13093244 34579120 PMC8466312

[4] Guiding Stars Licensing Company. How it works. Guiding Stars Simplifies Healthy eating.: Guiding Stars Licensing Company. (2022). Available online at: https://guidingstars.com/what-is-guiding-stars/ (accessed May 4, 2022).

[5] Volkova E Ni Mhurchu C. the influence of nutrition labeling and point-of-purchase information on food behaviours. Curr Obes Rep. (2015) 4:19–29. 10.1007/s13679-014-0135-6 26627087

[6] Sacks G Rayner M Stockley L Scarborough P Snowdon W Swinburn B. Applications of nutrient profiling: potential role in diet-related chronic disease prevention and the feasibility of a core nutrient-profiling system. Eur J Clin Nutr. (2011) 65:298–306. 10.1038/ejcn.2010.269 21245876

[7] mpconsulting. Health Star Rating System Five Year Review Report. Melbourne (2019).

[8] Storcksdieck Genannt Bonsmann S Marandola G Ciriolo E Van Bavel R Wollgast J. Front-of-pack nutrition labelling schemes: a comprehensive review. EUR 29811 EN. Luxembourg: Publications Office of the European Union. (2020).

[9] Vandevijvere S Vermote M Egnell M Galan P Talati Z Pettigrew S. Consumers' food choices, understanding and perceptions in response to different front-of-pack nutrition labelling systems in Belgium: results from an online experimental study. Arch Public Health. (2020)78:30. 10.1186/s13690-020-00404-3 32266069 PMC7119293

[10] Garritty C Gartlehner G Nussbaumer-Streit B King VJ Hamel C Kamel C. Cochrane Rapid Reviews Methods Group offers evidence-informed guidance to conduct rapid reviews. J Clin Epidemiol. (2021) 130:13–22. 10.1016/j.jclinepi.2020.10.007 33068715 PMC7557165

[11] Amir-Behghadami M Janati A. Population, Intervention, Comparison, Outcomes and Study (PICOS) design as a framework to formulate eligibility criteria in systematic reviews. Emerge Med J. (2020) 37:387. 10.1136/emermed-2020-209567 32253195

[12] International Organization for Standardization. ISO 26642:2010. Food products – determination of the Glycaemic Index (GI) and recommendation for food classification. (2010). Available online at: https://www.iso.org/standard/43633.html (accessed November 29, 2021).

[13] FoodSwitch: The George Institute for Global Health. Available online at: https://www.georgeinstitute.org/projects/foodswitch (accessed May 21, 2021).

[14] AUSNUT 2011-2013 food nutrient database: Food Standards Australia New Zealand; Available online at: https://www.foodstandards.gov.au/science/monitoringnutrients/ausnut/ausnutdatafiles/Pages/foodnutrient.aspx (accessed May 21, 2021).

[15] Merlin T Weston A Tooher R Middleton P Salisbury J Coleman K. NHMRC levels of evidence and grades for recommendations for developers of guidelines. National Health and Medical Research Council. (2009).

[16] Healthy Food Partnership. Partnership Reformulation Program: Food categories and reformulation targets. Healthy Food Partnership (2021).

[17] Food Standards Australia New Zealand. Australia New Zealand Food Standards Code – Schedule 4 – Nutrition, health and related claims: FSANZ. (2017).

[18] Healthy Food Partnership. Healthy Food Partnership Reformulation Program: Evidence Informing the Approach, Draft Targets and Modelling Outcomes. (2018).

[19] Chantal J Hercberg S. Development of a New Front-of-Pack Nutrition Label in france: The Five-Colour Nutri-Score. Copenhagen: World Health Organization Regional Office for Europe (2017). Avaialble online at: https://apps.who.int/iris/handle/10665/325207.

[20] Rayner M Scarborough P Lobstein T. The UK Ofcom Nutrient Profiling Model. Defining ‘Healthy' and ‘Unhealthy' Foods and Drinks for TV Advertising to Children. London: Food Standards Agency (2009).

[21] Rayner M Scarborough P Stockley L Boxer A. Nutrient Profiles: Development of Final Model. London: Food Standards Agency (2005).

[22] Neuenschwander M Ballon A Weber KS Norat T Aune D Schwingshackl L. Role of diet in type 2 diabetes incidence: umbrella review of meta-analyses of prospective observational studies. BMJ. (2019) 366:l2368. 10.1136/bmj.l2368 31270064 PMC6607211

[23] Reynolds A Mann J Cummings J Winter N Mete E Te Morenga L. Carbohydrate quality and human health: a series of systematic reviews and meta-analyses. Lancet. (2019) 393:434–45. 10.1016/S0140-6736(18)31809-9 30638909

[24] Binia A Jaeger J Hu Y Singh A Zimmermann D. Daily potassium intake and sodium-to-potassium ratio in the reduction of blood pressure: a meta-analysis of randomized controlled trials. J Hypertens. (2015) 33:1509–20. 10.1097/HJH.0000000000000611 26039623

[25] Cai X Li X Fan W Yu W Wang S Li Z. Potassium and obesity/metabolic syndrome: a systematic review and meta-analysis of the epidemiological evidence. Nutrients. (2016) 8:183. 10.3390/nu8040183 27023597 PMC4848652

