# Supplementary material for: Methodology for the Review and Update of Nutrient Criteria Underpinning Front-of-Pack Labeling: Application to the Glycemic Index Symbol
Source: Front Nutr. 2022 Jun 9;9:867349. doi: 10.3389/fnut.2022.867349 (PMC9218624; doi:10.3389/fnut.2022.867349)
Supplement: Supplementary file 1 [file Data_Sheet_1.docx]

Supplementary Material

**Supplementary Methods S1.** Summary of literature review findings and results of modelling for update of the GI Symbol PENC.

1. **Summary of literature review findings.**

*Carbohydrate quality*

The carbohydrate quality measures of glycemic load (1-3) and fibre (4, 5) showed strong and consistent associations with several health outcomes, including type 2 diabetes, and were deemed to be easy to apply to the modelling. Both absolute fibre and the carbohydrate to fibre ratio were relevant to the inclusion of fibre as a nutrient within the updated criteria. The carbohydrate to fibre ratio is a metric used to identify healthier grain products, proposed by the American Heart Association in 2010 (6), with foods meeting a carbohydrate to fibre ratio ≤ 10:1 associated with improved health outcomes and increased nutritional quality (6-8). While whole grain intake was associated with a number of benefits for health, including type 2 diabetes and CVD (9-11), whole grains were excluded from the modelling due to being difficult to model, with not all products reporting whole grain content in their ingredients list. Starch was excluded from the modelling due to a limited amount of evidence identified for total starch and chronic disease risk.

*Additional measures*

The additional nutritional measures of total sugars, added sugars, sodium, saturated, unsaturated and trans fatty acids, and protein were included in the modelling based on the strength of the scientific evidence and applicability to the food industry. While added sugars were deemed to be more difficult to model, they were found to be better predictors of nutritional quality for some food groups compared to total sugars, for example, in sugar-sweetened beverages, where an association with negative health outcomes and lower diet quality was identified (4, 5).

Both absolute saturated fat and the unsaturated to saturated fat ratio were deemed relevant to the inclusion of saturated fat as a criterion. The unsaturated to saturated fat ratio was found to be a stronger predictor of health outcomes, primarily CVD risk, compared to total fat (6). Energy was excluded due to the energy density of individual foods being primarily determined by total fat and water content, which could unfairly select against high fat, low water reformulations containing healthy, whole foods such as nuts. Processing level was excluded due to the possible arbitrary nature of food processing classification systems (7-9), making implementation into nutrient criteria difficult. While potassium was found to be an important nutrient for health outcomes, particularly with respect to the sodium to potassium ratio (10, 11), potassium was excluded due to the current lack of potassium as a mandatory nutrient on the nutrition information panel of food products, creating an added barrier for industry.

1. **Summary of modelling results.**

*General*

As per the overall guidelines of the GI Symbol PENC, foods were required to have a GI of ≤ 55 to qualify to be considered for GI Symbol certification or have a GI 25% lower than that of a reference food to be considered for ‘lower GI’ certification. Of the 56 food categories, 7 were deemed to be not feasible for reformulation, resulting in the modelling of 49 individual food categories.

*Carbohydrate quality*

Carbohydrate quality measures were based on a food category being grain based or not; a carbohydrate to fibre ratio of ≤ 10:1 was applied to grain food categories only, based on the development of the carbohydrate to fibre ratio for this purpose, whereas glycemic load was applied to non-grain based food categories. The inclusion of carbohydrate within the carbohydrate to fibre ratio made application of glycemic load redundant in grain-based food categories. Glycemic load was set as ≤ 33 for meals (three meals per day with a total recommended daily GL of ≤100), ≤ 25 for non-dairy core beverages, core grains and cereals, vegetables, nuts, and legumes (based on being major carbohydrate sources and four servings per day with a total recommended daily GL of ≤100), ≤ 19 for discretionary foods and beverages (to provide a medium GI), and core fruits and dairy, and ≤ 10 for spreads (to provide a low GI). Following application of the carbohydrate to fibre ratio or glycemic load, a minimum level for fibre content in g per serve was applied to food categories with outlying low levels of fibre, as well as food categories serving as meal replacements. The exception was ‘electrolyte sports drinks’, to which total available carbohydrate was applied to reflect the unique technical specifications of this food category.

*Additional measures*

Following the application of carbohydrate quality measures, additional nutritional measures were applied to both grain based and non-grain based food categories, on a food category specific basis. For some grain-based food categories, the carbohydrate to fibre ratio was sufficient to remove high levels of total sugars within relevant food product. A total sugars limit was applied to food categories having a current HFP target for total sugars (12), or containing added sugars as the major sugar source and having outlying levels of total sugars. Added sugars was deemed necessary in some instances to improve specificity. This included sugar-sweetened beverages, and food categories high in total sugars from both natural and added sources, such as canned fruit, dried fruit, and dried fruit products, where a total sugars limit would not differentiate between natural and added sugars. The added sugars limit was set based on the mean level of added sugars within each food category, with the aim to remove products with outlying high levels of added sugars. Sodium was applied to all food categories with a current HFP target for sodium (12), a sodium limit within the current PENC, or elevated outlying sodium levels. Given the evidence of replacing saturated fat with unsaturated fat for health (13-17), a ratio of unsaturated to saturated fat was applied to foods not meeting a designated saturated fat limit, defined by the current HFP target for saturated fat (12), or saturated fat limit within the current PENC. The World Health Organisation has recommended targets of ≤10% energy for saturated fat, and ≤35% energy for total fat (18), reflecting an unsaturated to saturated fat ratio limit of ≥ 2.5:1. There was also evidence that saturated fat from dairy foods have a different relationship to cardiovascular health risk factors, compared to other saturated fat sources (19, 20). Thus, an unsaturated to saturated fat limit of 2.5:1 was applied to non-dairy food categories, and an unsaturated to saturated fat limit of 0.5:1 was applied to dairy food categories or food categories containing dairy fat as the primary fat source, based on the ratio of unsaturated fat to saturated fat occurring naturally in whole milk. Trans fat limits were applied to food categories with a trans fat limit within the current PENC or found to be a major source of trans fat in the diet via the literature (18) or levels apparent within the AUSNUT database, with the limit set as the average level of trans fat within each relevant food category. A minimum protein limit was applied to food categories intended to serve as protein sources or meal replacements. This included toddler food categories, with the minimum level of protein set relative to that used for adult food categories, based on the relative Recommended Dietary Intake (RDI) for protein in children aged 1-3 years being approximately 25% of the average RDI for protein in adults. Based on stakeholder recommendation, a minimum calcium limit was applied to food categories intended to serve as dairy milk replacements.

**Supplementary Table S1.** Data extraction table used for comprehensive literature review (Phase III).

| **Nutritional measure** | **Authors (year)** | **Study design** | **Included studies, n (for SLR/MA)** | **Population details** | **Health outcomes and direction of effect** | **Dose-response information** | **Food-specific information** |
| --- | --- | --- | --- | --- | --- | --- | --- |
| *Eg. total vs added sugars* | *Starck (2019)* | *SLR* | *RCT, 12* | *Healthy children, Australia* | *50% reduction in postprandial blood sugar* | *None* | *Increased negative effect for added sugars in sweetened beverages* |
|  |  |  |  |  |  |  |  |

**Supplementary Table S2.** Full list of GI Symbol PENC food categories and their definitions.

| **Food category** | **Food sub-category** | **Description** |  |
| --- | --- | --- | --- |
|  |  |  |  |
|  |  |  |  |
| Cereal Grains and Products | Breads and Crispbreads | All breads and crispbreads, including rice/corn cakes, but not including crackers. |  |
|  | Breakfast Cereals | Includes muesli, bran-based cereals, cereal clusters, oat-based cereals, wheat biscuits (such as Weet-Bix), wheat flakes, etc. |  |
|  | Breakfast Biscuits | Biscuit-type products designed to be eaten as a breakfast replacement e.g., Belvita, Uncle Toby’s breakfast bakes. Does not include wheat-based biscuits such as Weet-Bix. |  |
|  | Bakery Products | Includes cakes, muffins, slices, fruit pies, pikelets, pancakes, crumpets, waffles, hotcakes, and sweet biscuits (fresh, frozen or made from packet mix). |  |
|  | Muesli Bars | All grain and cereal based bars e.g., oats, quinoa, wheat; with or without nuts and dried fruit. Includes applicable breakfast bars. |  |
|  | Plain Grains and Pasta | All plain grains, as well as plain pasta e.g., bran, oats, pasta, noodles, rice, couscous, polenta, wheat, barley, burghul, tapioca, sago, quinoa. |  |
|  | Grain and Pasta Products | Filled Pasta (e.g., ravioli), Instant/Savoury Noodles, Combined Pasta and Sauce Mixes, savoury and flavoured rice, quinoa or other grains served as an accompaniment to main meals e.g., rice/grain and vegetable mixes, microwave flavoured rice. Nutrient limits apply to the cooked products, ready for consumption. |  |
| Nuts, Legumes and other Plant-based protein alternatives | Nuts and Seeds - plain, unsalted | All plain nuts and seeds where nuts and seeds are the only ingredients. Does not include roasted or salted varieties. |  |
|  | Nuts and Seeds - coated/salted/flavoured | Plain or roasted nuts and seeds with added salt, oils, fats, and other flavourings. |  |
|  | Dried legumes | Plain dried legumes with no additional ingredients. |  |
|  | Shelf-stable legumes | Canned or vacuum-packed legumes e.g., canned chick-peas, baked beans. |  |
|  | Plant-based protein alternatives | All plant-based animal protein alternatives, including soy products e.g., tofu, tempeh and textured vegetable protein-based products, veggie burgers, 'not-chicken' burgers |  |
| Fresh Fruit and Fruit Products | Dried fruit processed (added sugar, fat, salt) | Dried fruit with added sugars, fats, salt, and other flavourings. |  |
|  | Canned fruit | Canned fruit in juice or with syrup. |  |
|  | Fresh, dried and frozen fruit | Plain fresh, dried and frozen fruit with no additions. |  |
|  | Dried Fruit Bars | Dried fruit bars, fruit straps and fruit bites (such as fruit-based bliss balls). |  |
|  | Fruit and Nut Mixes, processed | Combination of dried fruit (typically sultanas, currants or raisins) and nuts, such as trail mix, Scroggin, Gorp. |  |
| Fresh Vegetables and Vegetable Products | Fresh, frozen or dried vegetables | Plain fresh, dried and frozen vegetables with no additions. |  |
|  | Frozen or Dried Vegetable products | Frozen or dried vegetables with additions, as prepared. |  |
|  | Canned Vegetables with or without sauce | Canned vegetable products with or without added sauce. |  |
|  | Frozen potato products | Includes products such as frozen fries, hash browns, wedges, croquettes etc. |  |
| Milk, Dairy Products and Alternatives | Milk Fluid, Dried - plain | Plain dairy milk, including milk powder and lactose-hydrolysed milk. |  |
|  | Flavoured Milk and Dairy Drinks (including beverage mixes), as prepared | All dairy-based drinks with added flavourings and sugars, including chocolate/strawberry/banana milk and iced coffee beverages, and beverage mixes. |  |
|  | Non-Dairy Beverage Alternatives - Plain | Unflavoured plant-based milks including soy, oat, almond, coconut milk for drinking etc. |  |
|  | Non-Dairy Beverage Alternatives - Flavoured | Plant-based (including milks including soy, oat, almond, coconut milk for drinking etc) milks with added flavourings and/or sugars. |  |
|  | Evaporated Milk | All evaporated milks. |  |
|  | Frozen/Chilled Dairy Products, iced confection | Frozen dessert, ice cream, frozen yoghurt, gelato, sorbet, mousse, custard, and frozen ice products e.g., Streets Splice; includes non-dairy alternatives. |  |
|  | Yoghurt - plain unsweetened | Plain, unsweetened yoghurt, including plain lactose-hydrolysed varieties. |  |
|  | Yoghurt - sweetened | Includes all dairy and dairy-free yogurt alternatives like coconut/almond yogurt, as well as fromage frais, and toddler yoghurt/custard foods. |  |
| Snack Foods | Savoury Snacks | Includes popcorn, potato crisps, extruded snacks, soy chips, crackers, savoury nut-based snacks |  |
|  | Sweet Snacks | Sweet snacks that do not fit into the muesli bar or dried fruit product categories. Includes sweet nut and seed-based snacks/bars, chocolate bars, etc. |  |
| Sports Drinks and Sports Bars | Sports Drinks - electrolyte | Drinks designed to replace electrolytes lost through sweat during sport/physical activity. |  |
|  | Sport drinks - protein | Drinks designed to deliver a high amount of protein as a recovery from sports/physical activity. |  |
|  | Sports Bars and Miscellaneous Sports Products | Protein and energy bars and other non-beverage products designed for sporting use. |  |
| Special purpose foods | Formulated meal replacements | All that meet the criteria outlined in Food Standard 2.9.3. e.g., Sustagen. |  |
|  |  |  |  |
|  | Formulated supplementary foods | All that meet the criteria outlined in Food Standard 2.9.3. e.g., Milo. |  |
|  | Formulated supplementary foods for young children | All that meet the criteria outlined in Food Standard 2.9.3. e.g., Infant formula. |  |
|  | Formulated supplementary sports foods | All that meet the criteria outlined in Food Standard 2.9.4. e.g., Sports energy gel. |  |
|  | Foods for special medical purposes | All that meet the criteria outlined in Food Standard 2.9.5. e.g., Glucerna. |  |
| Beverages | Liquid Breakfasts | Beverages designed to be a replacement for breakfast e.g., Up and Go. |  |
|  | 100% fruit and vegetable juice | 100% fruit and/or vegetable juice. 1 serve = 125 mL, to align with the dietary guidelines. Includes 100% fruit and vegetable juice blends. |  |
|  | Other sweetened beverages | Includes fruit juices and drinks containing less than 100% juice, and with added sugars and/or other flavours, and any other water-based beverages containing added carbohydrate, e.g., flavoured waters, Kombucha, coconut water, tonic shots. |  |
| Convenience Foods | Soups (as prepared) | Includes powdered, concentrated and ready-to-eat varieties. Criteria apply to product as prepared. |  |
|  | Prepared Salads | Pre-prepared commercial salads containing a source carbohydrate. E.g., potato, bean or pasta-based |  |
|  | Pre-prepared/Ready to eat (RTE) meals | Frozen, canned or fresh meals that have been pre-prepared and are bought ready to eat, including pasta dishes, casseroles with rice/potato, curry and rice, stir-fry meals and rice, TV dinners and products containing a mixture of seafood and carbohydrate (e.g., tuna and rice/beans). |  |
|  | Other convenience foods | Other pre-prepared foods that are not designed as a ready to eat meal. E.g., meat pies, pasties, sausage rolls, pizza, filled wraps, sandwiches and rolls, quiche/frittata-type products etc |  |
|  | Coated seafood products | Processed products containing seafood and a source of carbohydrate e.g., fish fillets, fish fingers, fish cakes, coated with breadcrumbs or equivalent. |  |
| Toddler Foods (12-24 months of age) | Toddler RTE meals | All pre-prepared ready-to-eat toddler main meals containing meat, vegetables and starches. |  |
|  | Toddler breakfasts | Pre-prepared toddler-focused oat and muesli products. |  |
|  | Toddler pureed fruit | Fruit puree-based pouches made for toddlers |  |
|  | Toddler finger foods | Snack bars, fruit puffs made for toddlers |  |
|  | Toddler desserts | Chocolate, custard, and yoghurt products/pouches designed for toddlers |  |
| Sauces, Spreads and Dips | Sauces and Savoury Condiments | Used as additions and flavourings e.g., pasta, cook-in sauces, HP sauce, tomato sauce, chutney, relish, pickle, etc. |  |
|  | Sandwich Spreads - Nut-based | E.g., peanut butter, Nutella. Does not include yeast-based spreads. |  |
|  | Sandwich spreads - Sweet | E.g., honey, jam, marmalade. |  |
|  | Dips | E.g., hummus, sour-cream based dips, guacamole. |  |

Abbreviations: PENC, Product Eligibility and Nutrient Criteria; GI, Glycemic Index.

**Supplementary Table S3.** Candidate food composition databases identified for modelling, with detail on characteristics per each criteria used for selection.

| **Food composition database** | **Characteristics** | | | | | | |  |  |
| --- | --- | --- | --- | --- | --- | --- | --- | --- | --- |
|  | **Availability** | **Up-to-date** | **Representative of all products** | **Data for additional nutritional components** | **Format** | **Previous use in modelling** | | |  |
| Coles/Woolworths-owned | No | NA | Yes; all products available in supermarket. | NA | NA | | NA | | |
| FoodSwitch | Available at cost | Most recent. | Yes | Added and free sugars available at increased cost; whole grain percentage in some ingredient list where applicable. | Excel workbook | | Yes; particularly used for HSR assessment (21). | | |
| AUSNUT | Free and publicly available | No, published 2011-2012 | No, individual foods placed into larger generic categories. | Added sugars, free sugars, and trans-fat; no wholegrain information. | Excel workbook | | Yes; used in published diet quality and cost modelling (22). | | |
| Contract | Available at cost | Data older than FoodSwitch | Yes | NA | NA | | Yes; applications presented at international and national conferences (23). | | |
| Abbreviations: HSR, Health Star Rating; NA, information not available. | | | | | | | | | |

**Supplementary Table S4.** Comparison of tools for assessment of updated PENC.

| **Nutritional quality assessment tool** | **Purpose of tool** | **Characteristic for comparison** | | | | |  |
| --- | --- | --- | --- | --- | --- | --- | --- |
|  |  | **Correlation of food categories with GI Symbol categories** | **Nutrients covered** | **Food-based criteria** | **Correlation of nutrient criteria with current GI Symbol criteria** | **Is all required information available?** | **Will the criteria be easy to apply?** |
| Health Star Rating | To highlight healthier packaged food products. | Broader food categories; easy correlation. | Energy, saturated fat, total sugars, sodium, protein, fibre. | Fruit, vegetables, nuts and legumes (FVNL). | Yes, except for total sugars and FVNL. | Yes; calculator free to download. | Yes |
| Healthy Food Partnership | To create targets for the reformulation of manufactured foods, that will improve the overall healthiness of the food supply. | 18 food categories, with some divided into sub-categories to create 41 categories overall; correlate well with GIF categories. | Saturated fat, total sugars, sodium | None | Easily correlates; however only three nutrients included so unlikely to indicate performance. | Yes - freely available online | Yes. |
| Healthy Kids Association | To assist consumers to easily identify ‘Better Choice’ foods and beverages for children aged 5-17 years, within a food category. | 51 food categories. Very similar to GIF food categories. Also includes non-CHO products. | Energy, added sugars, sodium, saturated fat, total fat, protein, calcium, fibre. | Whole-foods, processed meat, added confectionary, artificial sweeteners. | Appears to correlate well with GIF criteria, although is more stringent, so may not be feasible for industry to reach. | Yes. | Complicated: more food categories, each with more criteria per food category. |
| Diabetes AUS |  | Unknown | Unknown | Unknown | Unknown | No - we have none of the required information | Unknown |

Abbreviations: FVNL, fruit, vegetables, nuts, and legumes; CHO, carbohydrate; GI, Glycemic Index .

**Supplementary Table S5.** Aggregation of food-based recommendations for each of the nutritional measures.

| **Nutritional measure** | **Food-based recommendations** | | |
| --- | --- | --- | --- |
|  | **FSANZ** | **HFP** | **Additional** |
| *Carbohydrate quality* |  |  |  |
| Glycemic load (GL) | NA | NA | - Low GL ≤ 10; Medium GL 11–19; High GL ≥20^1^ - Total daily GL should be ≤ 100^2^ |
| Fibre | - Source of fibre, ≥ 2 g per serve; good source of fibre, ≥ 4 g per serve; excellent source ≥ 7 g per serve^3^. | NA | - Carbohydrate to fibre ratio^4^: a metric used to identify healthier grain products, proposed by the American Heart Association in 2010. - Foods with a carbohydrate to fibre ratio ≤ 10 have been associated with improved health outcomes and increased nutritional quality^4^. |
| *Additional nutritional measures* | |  |  |
| Total sugars | NA | Current HFP targets: flavoured dairy milk, 9g/100mL; flavoured alternative milks, 5g/100mL; flavoured water, 5g/100mL; fruit drinks, 9.5g/100mL; sweetened yoghurts: 12.5g/100g^5^. | Included within the carbohydrate to fibre ratio^4^ |
| Added sugars | NA | NA | NA |
| Sodium | NA | Current HFP targets: breads and crispbreads, 270-450 mg/100g; breakfast cereals, 270-450 mg/100g; cakes, muffins, and slices, 360 mg/100g; savoury snacks, 360-720 mg/100g; soups, 280 mg/100g; pizza, 400 mg/100g^5^. | NA |
| Saturated fat | NA | Current HFP targets: pizza, 4g/100g; pastries: 7 g/100g^5^. | NA |
| Unsaturated to saturated fat ratio | NA | NA | Australian recommended maximum intakes^6^: total fat, 35% dietary energy; saturated fat, 10% dietary energy; result in an unsaturated to saturated fatty acid ratio of 2.5:1. The unsaturated to saturated fatty acid ratio naturally present in dairy milk is 0.5:1. |
| Trans fat | NA | NA | NA |
| Protein | Source of protein, ≥ 5 g per serve; good source of protein (≥ 10 g per serve) ^3^. | NA | NA |

References cited are as follows:

^1^ [23, 24]

^2^ [25, 26]

^3^ [15]

^4^ [27-29]

^5^ [14]

^6^ [30]

NA, no recommendations identified

**Supplementary Methods S1.** Summary of stakeholder feedback for the updated GI Symbol PENC.

**Industry stakeholders**

The major feedback showing consistency across most, if not all, industry stakeholders was:

- Concern when specific food products failed to meet the criteria by a small margin only.
- Lack of clarity about rationale used to develop the criteria.
- Confusion about foods included within each food category due to a lack of definition.

**Academic stakeholders**

The major feedback from academic stakeholders was:

- Literature review: recommendations for additional evidence supporting nutritional components and grammatical revisions.
- Guiding principles: alignment of criteria with International Standards.
- Modelling methodology: clarification around rationale underpinning selected thresholds, and suggestion of use of added sugars for specific food categories.

1. Fontanelli MM, Micha R, Sales CH, Liu J, Mozaffarian D, Fisberg RM. Application of the <= 10:1 carbohydrate to fiber ratio to identify healthy grain foods and its association with cardiometabolic risk factors. European Journal of Nutrition. 2020;59(7):3269-79.

2. Liu J, Rehm CD, Shi P, McKeown NM, Mozaffarian D, Micha R. A comparison of different practical indices for assessing carbohydrate quality among carbohydrate-rich processed products in the US. PLOS ONE. 2020;15(5):e0231572.

3. Tan D, Olden AN, Orengo A, Francey C, Campos VC, Fayet-Moore F, et al. An Assessment of Three Carbohydrate Metrics of Nutritional Quality for Packaged Foods and Beverages in Australia and Southeast Asia. Nutrients. 2020;12(9):2771.

4. Sigala DM, Stanhope KL. An Exploration of the Role of Sugar-Sweetened Beverage in Promoting Obesity and Health Disparities. Current Obesity Reports. 2021;10(1):39-52.

5. Malik VS, Popkin BM, Bray GA, Després J-P, Hu FB. Sugar-sweetened beverages, obesity, type 2 diabetes mellitus, and cardiovascular disease risk. Circulation. 2010;121(11):1356-64.

6. Zhu Y, Bo Y, Liu Y. Dietary total fat, fatty acids intake, and risk of cardiovascular disease: a dose-response meta-analysis of cohort studies. Lipids in Health & Disease. 2019;18(1):91.

7. Labonté M-È, Poon T, Mulligan C, Bernstein JT, Franco-Arellano B, L'Abbé MR. Comparison of global nutrient profiling systems for restricting the commercial marketing of foods and beverages of low nutritional quality to children in Canada. The American Journal of Clinical Nutrition. 2017;106(6):1471-81.

8. Bleiweiss-Sande R, Chui K, Evans EW, Goldberg J, Amin S, Sacheck J. Robustness of Food Processing Classification Systems. Nutrients. 2019;11(6).

9. Drewnowski A, Gupta S, Darmon N. An Overlap Between “Ultraprocessed” Foods and the Preexisting Nutrient Rich Foods Index? Nutrition Today. 2020;55(2):75-81.

10. Binia A, Jaeger J, Hu Y, Singh A, Zimmermann D. Daily potassium intake and sodium-to-potassium ratio in the reduction of blood pressure: a meta-analysis of randomized controlled trials. Journal of Hypertension. 2015;33(8):1509-20.

11. Cai X, Li X, Fan W, Yu W, Wang S, Li Z, et al. Potassium and Obesity/Metabolic Syndrome: A Systematic Review and Meta-Analysis of the Epidemiological Evidence. Nutrients. 2016;8(4):183.

12. Healthy Food Partnership. Partnership Reformulation Program: Food categories and reformulation targets. Healthy Food Partnership,; 2021.

13. Schwab U, Reynolds AN, Sallinen T, Rivellese AA, Risérus U. Dietary fat intakes and cardiovascular disease risk in adults with type 2 diabetes: a systematic review and meta-analysis. Eur J Nutr. 2021.

14. Schwingshackl L, Zähringer J, Beyerbach J, Werner SW, Heseker H, Koletzko B, et al. Total Dietary Fat Intake, Fat Quality, and Health Outcomes: A Scoping Review of Systematic Reviews of Prospective Studies. Ann Nutr Metab. 2021;77(1):4-15.

15. Hooper L, Martin N, Abdelhamid A, Davey Smith G. Reduction in saturated fat intake for cardiovascular disease. Cochrane Database of Systematic Reviews. 2015(6):CD011737.

16. Wang DD, Li Y, Chiuve SE, Stampfer MJ, Manson JE, Rimm EB, et al. Association of Specific Dietary Fats With Total and Cause-Specific Mortality. JAMA Intern Med. 2016;176(8):1134-45.

17. Clifton PM, Keogh JB. A systematic review of the effect of dietary saturated and polyunsaturated fat on heart disease. Nutrition Metabolism & Cardiovascular Diseases. 2017;27(12):1060-80.

18. World Health Organisation. WHO draft guidelines on saturated fatty acid and trans-fatty acid intake for adults and children. Draft for public consultation.: World Health Organisation; 2018.

19. Duarte C, Boccardi V, Amaro Andrade P, Souza Lopes AC, Jacques PF. Dairy versus other saturated fats source and cardiometabolic risk markers: Systematic review of randomized controlled trials. Crit Rev Food Sci Nutr. 2021;61(3):450-61.

20. Guo J, Astrup A, Lovegrove JA, Gijsbers L, Givens DI, Soedamah-Muthu SS. Milk and dairy consumption and risk of cardiovascular diseases and all-cause mortality: dose-response meta-analysis of prospective cohort studies. Eur J Epidemiol. 2017;32(4):269-87.

21. Health Star Rating System Five Year Review Report. 2019.

22. Starck C, Blumfield M, Keighley T, Roesler A, Abbott K, Cassettari T, et al. Diet and economic modelling to improve the quality and affordability of the Australian diet for low and medium socioeconomic households. Int J Environ Res Public Health. 2021:[under review].

23. CSIRO. FoodTrack™ food and nutrient database: CSIRO; [Available from: <https://www.csiro.au/en/research/health-medical/nutrition/foodtrack>.

24. Food Standards Australia New Zealand. Australia New Zealand Food Standards Code – Schedule 4 – Nutrition, health and related claims.: FSANZ; 2017.
